# Prospective Mental Imagery in Depression: Impact on Reward Processing and Reward-Motivated Behaviour

**DOI:** 10.32872/cpe.3013

**Published:** 2021-06-18

**Authors:** Fritz Renner, Jessica Werthmann, Andreas Paetsch, Hannah E. Bär, Max Heise, Sanne J. E. Bruijniks

**Affiliations:** 1 Clinical Psychology and Psychotherapy Unit, Institute of Psychology, University of Freiburg, Freiburg, Germany; Philipps-University of Marburg, Marburg, Germany

**Keywords:** prospective mental imagery, depression, reward processing, motivation, behavioural activation

## Abstract

**Background:**

Mental imagery has long been part of cognitive behavioural therapies. More recently, a resurgence of interest has emerged for prospective mental imagery, i.e. future-directed imagery-based thought, and its relation to reward processing, motivation and behaviour in the context of depression.

**Method:**

We conducted a selective review on the role of prospective mental imagery and its impact on reward processing and reward-motivated behaviour in depression.

**Results:**

Based on the current literature, we propose a conceptual mechanistic model of prospective mental imagery. Prospective mental imagery of engaging in positive activities can increase reward anticipation and reward motivation, which can transfer to increased engagement in reward-motivated behaviour and more experiences of reward, thereby decreasing depressive symptoms. We suggest directions for future research using multimodal assessments to measure the impact of prospective mental imagery from its basic functioning in the lab to real-world and clinical implementation.

**Conclusion:**

Prospective mental imagery has the potential to improve treatment for depression where the aim is to increase reward-motivated behaviours. Future research should investigate how exactly and for whom prospective mental imagery works.

## Highlights

- This review provides a selected update of the literature on prospective mental imagery.
- Prospective mental imagery might decrease depression via reward processing and reward-motivated behaviours.
- Suggestions for future research to investigate these hypotheses are provided.

According to Beck’s cognitive model, individuals with depression hold negative views about the self, others and the future (Beck, Rush, Shaw, & Emery, 1979). In addition to the negatively biased content of future thinking in depression, the importance of thought modality, particularly mental representations, has increasingly been recognized as a key target in psychotherapy (Arntz, 2020). Thinking about events or activities in the future might draw on imagery-based thought, involving a rich perceptual experience in the absence of external sensory input (Pearson, Naselaris, Holmes, & Kosslyn, 2015). Prospective mental imagery, i.e. future-directed imagery-based thought, has recently gained interest in the context of depression. In this review, we provide a selected update of the recent scientific literature on prospective mental imagery and its impact on reward processing (i.e., anticipation or experience of reward) and reward-motivated behaviour (i.e., behaviour driven by the motivation to attain rewards) in depression. Drawing from the wider research in this area, we present a conceptual model linking prospective mental imagery to reward processing and reward-motivated behaviour and discuss future directions for research. For a broader discussion of the nature, function and clinical applications of mental imagery in depression and other mental disorders see e.g. Blackwell, 2019; Holmes, Blackwell, Burnett Heyes, Renner, and Raes, 2016; Ji, Kavanagh, Holmes, MacLeod, and Di Simplicio, 2019; Renner and Holmes, 2018.

## Identifying Core Clinical Features in Depression: Reward Processing

Major Depressive Disorder (MDD) is characterized by low mood and/or the loss of interest in previously rewarding or enjoyable activities as well as a number of other emotional, cognitive and physical symptoms (American Psychiatric Association, 2013). MDD is a heterogeneous disorder, meaning that two individuals with a diagnosis of MDD may have little or no symptoms in common (Strunk & Sasso, 2017). This presents a major challenge for research and treatment development in depression (Fried, 2015, 2017; Olbert, Gala, & Tupler, 2014). Accordingly, recent initiatives have called to focus research on core clinical features rather than psychiatric syndromes in depression and other mental disorders (Insel et al., 2010). Alterations in reward processing are common in psychopathology (Zald & Treadway, 2017) and therefore one potential treatment target in this context. In depression, alterations in reward processing might manifest in a reduced sensitivity to reward, resulting in decreased approach motivation (Alloy et al., 2016). Deficits in reward processing represent a central aspect of anhedonia, defined as “diminished interest or pleasure in almost all activities” (American Psychiatric Association, 2013). Diminished interest and diminished experienced pleasure correspond to two distinct components of reward processing: Reward anticipation and reward consummation (Gard, Germans Gard, Kring, & John, 2006; Treadway & Zald, 2011). Reward anticipation can be further divided into anticipated reward, i.e. the expectation of how rewarding/pleasant a future activity will be, and anticipatory reward, i.e. the subjective experience of how rewarding/pleasant it is to think about a future activity (Baumgartner, Pieters, & Bagozzi, 2008). Reward consummation, on the other hand, refers to rewarding/pleasant feelings experienced while engaging in enjoyable activities (Gard, Germans Gard, Kring, & John, 2006). While both components are important, research has suggested that deficits in reward-motivated behaviour are primarily driven by reduced or dysfunctional reward anticipation (Bakker et al., 2017; Gorka et al., 2014). Given that these deficits in reward processing are not adequately addressed by current treatments of depression (Treadway & Zald, 2011), one way forward in treatment innovation is to develop procedures directly targeting reward anticipation and reward-motivated behaviours.

## Targeting Reward Anticipation, Reward Motivation and Reward-Motivated Behaviours Using Prospective Mental Imagery

By drawing on shared brain structures and functions (Dijkstra, Bosch, & van Gerven, 2019; Kosslyn, Ganis, & Thompson, 2001; Pearson et al., 2015), vivid mental imagery can give rise to an “as real” experience and evoke emotional responses at subjective, physiological and neural levels (Ji, Burnett Heyes, MacLeod, & Holmes, 2016). These properties of prospective mental imagery allow us to simulate engagement in behavioural activities and to “pre-experience” future activities, thereby providing “a taste” of different courses of action and their potential (emotional) consequences (Moulton & Kosslyn, 2009). This makes prospective mental imagery an excellent candidate procedure to target reward anticipation and reward-motivated behaviours.

Recently, a number of studies have emerged that tested the impact of prospective mental imagery of positive events or activities on reward anticipation and reward-motivated behaviour. These studies have the common aim of investigating new ways of promoting positive experiences, in line with recent calls for treatment innovation in depression to focus on positive affect systems (Dunn, 2012; Dunn et al., 2019). Studies presented here also fit within the broader literature that highlights the role of expectancies in mental disorders (Rief & Glombiewski, 2017; Rief et al., 2015). In depression, an absence of positive expectancies might manifest as low anticipated reward/pleasure from engaging in otherwise enjoyable activities.

Several studies have investigated the impact of mental imagery on reward anticipation. In a case-series, Hallford, Sharma, and Austin (2020) asked participants with depression to rate anticipatory pleasure of future events over a no-treatment baseline phase. Participants then switched to an intervention phase in which they completed an episodic future thinking task involving vivid imagery of engaging in enjoyable upcoming activities, focussing on contextual and episodic detail of these events. The authors found large effects of the intervention on anticipatory pleasure. In two experimental studies, Hallford, Farrell, and Lynch (2020) further tested the impact of guided episodic thinking about past or future positive events on anticipated and anticipatory pleasure in a non-clinical sample. Participants were instructed to imagine past or future events from a first-person perspective emphasising positive aspects of the events. In general, the authors found support for their hypothesis that guided episodic thinking of positive events (past- and future-oriented) increases anticipated and anticipatory pleasure (compared to baseline ratings). In an earlier study, Pictet et al. (2016) tested the effect of an imagery cognitive bias modification (CBM) procedure on depression, anhedonia and anticipatory and consummatory pleasure in individuals with depressive symptoms. They found positive effects of the CBM intervention involving imagery of positive everyday experiences (compared to a closely matched control condition) on anhedonia and anticipatory pleasure as well as a stronger increase in consummatory pleasure (compared to a waitlist control condition). This is in line with earlier findings by Blackwell et al. (2015), who found positive effects of an imagery CBM intervention (compared to an active control condition) on the anhedonia item of the Beck Depression Inventory. These studies suggest that imagery-based interventions might have merit in targeting reward-related processes in depression.

Other studies have focussed on the effects of mental imagery on approach motivation. For example, Linke and Wessa (2017) tested the effects of an online mental imagery training, compared to a waitlist control condition, on reward sensitivity and approach tendencies towards positive activities and edibles. During the imagery training, participants imagined the positive emotions, affirmative thoughts and pleasurable sensations associated with previously selected positive activities every second day over a two-week period. The authors found that the imagery training successfully increased reward sensitivity and faster approach tendencies for activities (Linke & Wessa, 2017). Another study tested the effects of a positive prospective imagery intervention for planned everyday enjoyable and routine activities in a non-clinical sample (Renner, Murphy, Ji, Manly, & Holmes, 2019). Participants first selected and planned activities following the procedures described in behavioural activation treatment for depression (Martell, Addis, & Jacobson, 2001). Participants in a motivational imagery condition then vividly imagined engaging in each of their planned activities. Participants in a no-imagery control condition planned the activities, but did not engage in the imagery exercise. The prospective imagery intervention increased anticipated pleasure/reward and motivation to engage in the activities, compared to the control condition. In two independent experiments, Boland, Riggs, and Anderson (2018) asked non-depressed and dysphoric participants to simulate positive events using vivid mental imagery. They found that event likelihood (i.e., how likely participants thought the event would happen to them in the future) for positive events increased following imagery simulation of the events compared to a neutral imagery control task. Taken together, these studies demonstrate that engaging in positive prospective mental imagery of everyday activities has an impact on reward processing and transfers to approach motivation for engaging in the simulated activities.

Finally, a number of studies have investigated the transfer of the motivating effect of mental imagery interventions to self-reported activity levels outside the lab. One study conducted a secondary analysis of a randomized controlled trial (Blackwell et al., 2015) to test the effects of a four-week positive mental imagery intervention on self-reported behavioural activation in individuals with major depressive disorder (Renner, Ji, Pictet, Holmes, & Blackwell, 2017). Participants randomized to the positive imagery condition showed a greater increase in self-reported behavioural activation over the study period, compared to participants randomized to a control condition (Renner et al., 2017). In line with these findings, Renner et al. (2019; reviewed above) found that positive mental imagery simulations of planned activities was associated with higher completion of activities that participants had previously been putting-off doing. Considering all types of activities, mental imagery led to a higher completion compared to a control group receiving activity reminder messages but not to a control group without reminder messages. Thus, while these preliminary findings need replication, they provide initial evidence that the positive effects of prospective mental imagery on approach motivation for rewarding activities might transfer to reward-motivated behaviour outside the laboratory.

The studies reviewed here suggest that positive prospective mental imagery of activities can facilitate reward anticipation, reward motivation and reward-motivated behaviour. This is clinically relevant given that reward anticipation deficits are not adequately addressed in current treatments of depression (Treadway & Zald, 2011). Drawing from this broader literature, in the following paragraph, we provide a conceptual model describing how prospective mental imagery could promote the engagement in reward-motivated behaviour and its clinical potential to impact mood and depressive symptoms.

## Mental Imagery as Motivational Amplifier: A Conceptual Model

**Figure 1:**
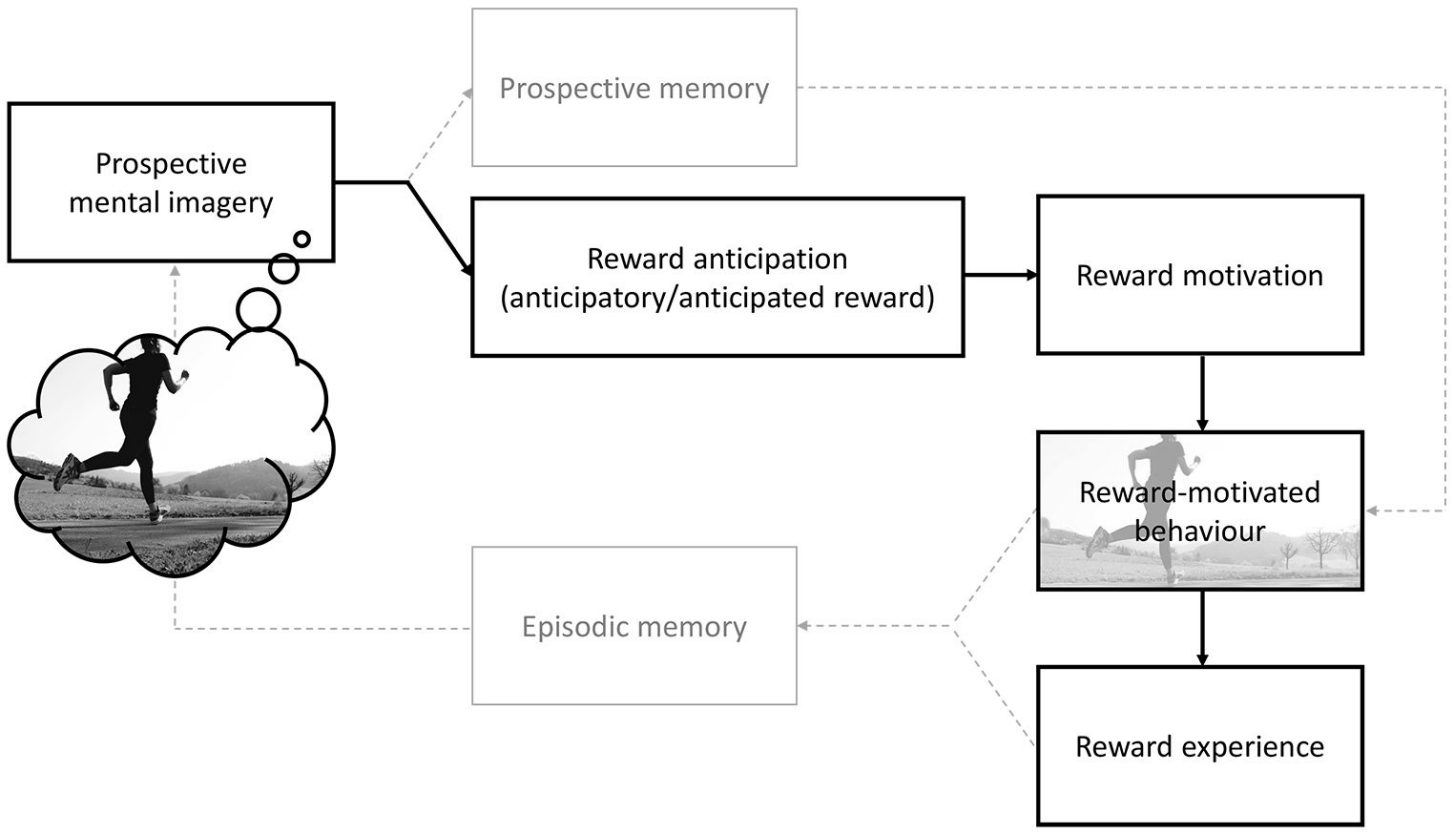
Conceptual Model of the Motivational Amplifier Hypothesis *Note.* Positive prospective mental imagery of engaging in (everyday) activities (e.g. running) can increase reward anticipation (anticipatory and anticipated reward) and reward motivation, which can transfer to increased engagement in reward-motivated behaviour and reward experience. Note that concepts in bold boxes are part of the literature review above.

Figure 1 provides a conceptual model illustrating the expected effects of prospective mental imagery on reward-motivated behaviour: positive prospective mental imagery of activities gives rise to a *motivational amplifier* effect by facilitating reward anticipation, reward motivation and reward-motivated behaviour. Given the power of mental imagery to amplify emotions (Holmes, Geddes, Colom, & Goodwin, 2008), it has the potential to evoke the anticipation of reward-related emotions by drawing upon prior knowledge and experiences (Kavanagh et al., 2005; Moulton & Kosslyn, 2009; Schacter et al., 2008). Anticipating the positive emotional consequences of future behaviour, in turn, predicts reward motivation and reward-motivated behaviour (Hallford & Sharma, 2019; Mellers & McGraw, 2001; Sherdell, Waugh, & Gotlib, 2012; Treadway & Zald, 2011). This transfer from imagery to behaviour might be further facilitated by a boost in prospective memory for the simulated activity (Schacter, Benoit, & Szpunar, 2017). Actual engagement in simulated activities might then lead to a reward experience. The episodic memory of this experience, in turn, affects subsequent imagery simulations of similar future activities (Figure 1, see Table 1 for key term definitions).

**Table 1:** Definition of Key Terms Used in the Conceptual Model

Concept	Definition
Prospective mental imagery	Future-directed imagery-based thought, involving a rich perceptual experience without external sensory input
Reward processing	
Reward anticipation	
Anticipated reward	Expectation of how rewarding/pleasant a future activity will be
Anticipatory reward	Subjective experience of how rewarding/pleasant it is to think about a future activity
Reward experience	Pleasant/rewarding feelings experienced while engaging in the activity
Reward motivation	Amount of effort an individual is prepared to expend for reward attainment
Reward-motivated behaviour	Behaviour driven by the motivation to attain rewards
Prospective memory	Remembering to carry out a planned activity in the future
Episodic memory	Memory of personal experiences

**Figure 2:**
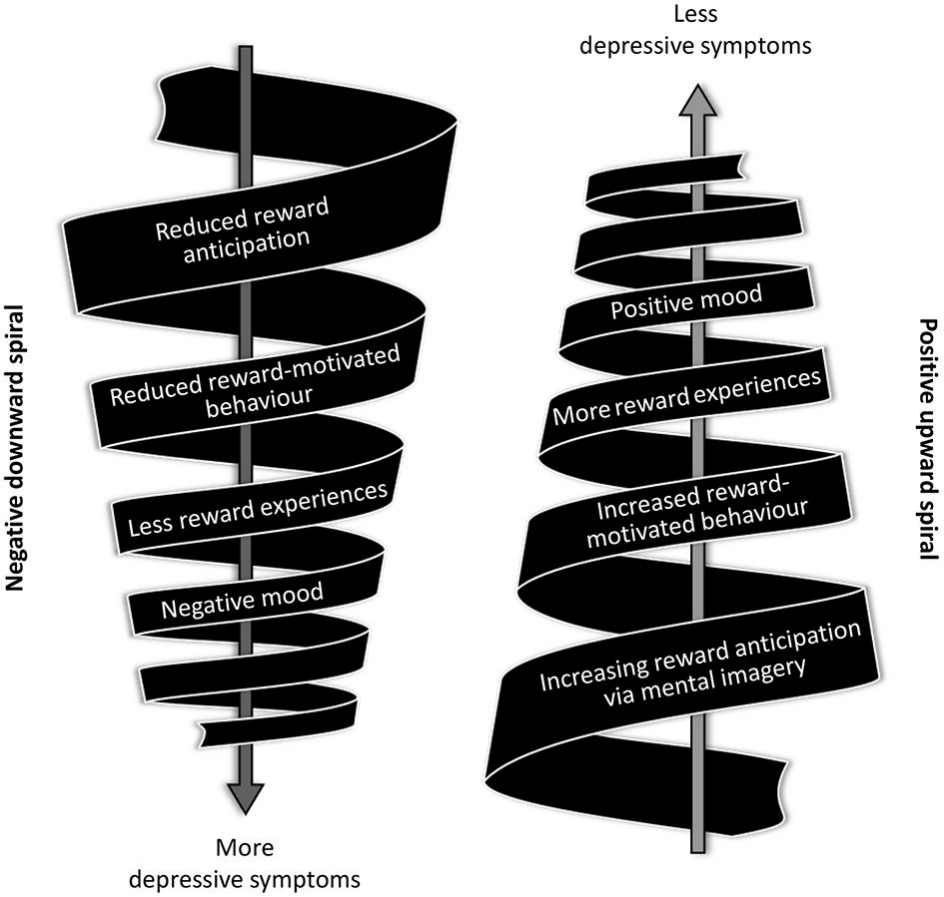
Reversing the Downward Spiral of Depression with Prospective Mental Imagery *Note.* Key assumption: Reduced reward anticipation leads to a downward spiral of reduced reward-motivated behaviour and less reward experiences, resulting in increased depressive symptoms (left side). Key hypothesis: Targeting reward anticipation using vivid prospective mental imagery leads to increased reward-motivated behaviour and more reward experiences, resulting in a decrease of depressive symptoms (right side).

The conceptual model has clinical potential insofar as it illustrates how positive prospective mental imagery could be used to promote behavioural activation in depression. The central assumption here is that reduced reward anticipation in depression contributes to a downward-spiral of reduced reward-motivated behaviours due to a loss of interest in previously rewarding activities that reduces the experience of rewards in daily life and, consequently, worsens depressive symptoms such as low mood (Figure 2). Based on our conceptual model, we hypothesise that positive prospective mental imagery of everyday activities can reverse this process by acting as a *motivational amplifier* boosting behavioural activation and thereby alleviating depressive symptoms (Figure 2).

In summary, the recent literature reviewed above supports the idea that positive prospective mental imagery of activities can facilitate reward anticipation, reward motivation and reward-motivated behaviour. However, the reviewed studies primarily relied on self-report and more work is needed to investigate how the transfer of imagery to behaviour beyond the laboratory can be facilitated and how prospective mental imagery might benefit clinical practice.

## Future Directions

Recent literature has emphasised the importance of conducting multimodal research to understand and thereby improve clinical interventions (Holmes, Craske, & Graybiel, 2014). A future endeavour might thus be to extend previous research on the mechanism underlying prospective mental imagery beyond self-report. Neuroimaging, for instance, has provided initial evidence for a recruitment of brain regions implicated in reward processing, such as the dorsal (caudate nucleus) and ventral striatum (nucleus accumbens), during prospective mental imagery of positive events (D’Argembeau, Xue, Lu, Van der Linden, & Bechara, 2008; Gerlach, Spreng, Madore, & Schacter, 2014). Other measures that have been used to evaluate imagery-based manipulations and reward processing include pupil size, attention bias and approach/avoidance tendencies (Anderson, Laurent, & Yantis, 2011; Henderson, Bradley, & Lang, 2018; Linke & Wessa, 2017; Schneider, Leuchs, Czisch, Sämann, & Spoormaker, 2018; Werthmann, Jansen, & Roefs, 2016). Similar approaches could prove useful to further investigate reward processing as a working mechanism of prospective mental imagery for behavioural activation. Ultimately, investigations beyond self-report will help us fine-tune imagery-based interventions and thereby guide treatment innovation for depression.

Another important question in experimental psychopathology research is how lab-based findings hold up under everyday circumstances. Recent research in the broader field of clinical psychology demonstrated the added value of combining laboratory experiments with Ecological Momentary Assessment (EMA; e.g. Bakker et al., 2019; Moran, Culbreth, & Barch, 2017; Ramirez & Miranda, 2014) and of integrating experimental manipulations into daily life (Huffziger et al., 2013; Huffziger, Ebner-Priemer, Koudela, Reinhard, & Kuehner, 2012). For example, Bakker and colleagues (2019) showed that when neural activity in reward processing regions was lower, assessed in the lab, EMA of reward anticipation and activity pleasantness were increasingly dissociated from one another. Findings like these can be valuable to refine or develop interventions by identifying treatment targets (e.g. coupling of anticipation and engagement) under well-specified circumstances (e.g. low neural activity in reward-processing brain regions). These findings are also relevant in the context of earlier findings regarding challenges with the transfer of experimental prospective mental imagery interventions from lab to the real world (Renner et al., 2019). Integration of EMA with lab-based experiments as well as the use of Ecological Momentary Interventions (EMI; Myin-Germeys, Klippel, Steinhart, & Reininghaus, 2016) or manipulations of reward processing through prospective mental imagery in daily life may offer an additional means to facilitate the transfer from lab to real-world behaviour.

Moreover, individuals may differ in the extent to which they benefit from prospective mental imagery interventions. Studies already pointed to individual variation in processes related to prospective mental imagery, such as anticipatory pleasure (Hallford, Sharma, & Austin, 2020) and the perception of reward (Locke & Braver, 2008), and suggested promising potential predictors or moderators that should be investigated in future studies. Potential moderators include individual differences in generating vivid mental imagery (Blackwell et al., 2015; Renner et al., 2017, 2019), procrastination (Renner et al., 2019) and the number of depressive episodes (Blackwell et al., 2015). Additionally, when moving towards clinical applications, the question of how individual differences interact with the active ingredients of prospective imagery interventions becomes relevant. For example, initial evidence highlights the importance of simulating rewarding aspects of planned activities in non-clinical participants, but it has not yet been investigated if individuals who have difficulties experiencing pleasure/reward from (thinking about) activities (i.e., individuals with anhedonia) benefit from simulating rewarding aspects of planned activities. Relatedly, prospective mental imagery interventions developed in the lab might need to be adjusted for clinical groups. For example, individuals with low mood and depression experience more difficulty in generating vivid prospective imagery and experience less spontaneous positive imagery (Hallford, Barry, et al., 2020; Holmes et al., 2016; Ji, Holmes, MacLeod, & Murphy, 2019; Morina, Deeprose, Pusowski, Schmid, & Holmes, 2011). Individuals with depression might thus benefit from additional training in generating vivid imagery for positive events.

Imagery based interventions have been used as stand-alone interventions as well as part of regular CBT for depression (Renner & Holmes, 2018). So far, we have mainly discussed the use of prospective mental imagery to target specific core clinical features in depression. Another line of inquiry involves integrating prospective imagery procedures to enhance the effects of established treatments for depression. Recent studies have suggested that CBT might be improved by the use of cognitive support strategies that enhance memory for the session content, and subsequently outcome (Harvey et al., 2017, 2014). We suggest that prospective mental imagery could potentially work as a cognitive support strategy for CBT skill acquisition. CBT skills have been defined as the ability to re-evaluate the accuracy of one's own automatic beliefs or underlying stable cognitive patterns (a cognitive therapy skill; CT skill) and to engage proactively in pleasurable activities (a behavioral therapy skill; BT skill) (Strunk, DeRubeis, Chiu, & Alvarez, 2007). In non-clinical settings, mental imagery has been linked to improved skill acquisition in health-related and sport contexts (Anton, Bean, Hammonds, & Stefanidis, 2017; Dana & Gozalzadeh, 2017). In a clinical setting, mental imagery has been indirectly linked to BT skill by demonstrating an impact on self-reported behavioural activation (Renner et al., 2017; reviewed above). Future studies should investigate how and for whom prospective mental imagery may increase the acquisition of CBT skills. Further down the road, for a successful clinical implementation, training sessions in prospective mental imagery could be included as part of a regular behavioural activation treatment protocol (Martell et al., 2001) to facilitate engagement in pleasant and rewarding activities.

## Overall Conclusion

In this review, we provided a selected update of the recent scientific literature on prospective mental imagery and its impact on reward processing and reward-motivated behaviour in depression. Overall, the studies presented here suggest that prospective mental imagery simulations of activities can increase reward processing related to these activities as well as reward motivation and reward-motivated behaviors. Thus, these initial studies suggest that prospective mental imagery is a promising experimental intervention in the context of depression, where the aim is to increase engagement in potentially rewarding activities. Future directions for research in this area may focus on multimodal assessments of prospective mental imagery effects to gain a better understanding of the processes involved, from basic mechanisms to everyday situations and its clinical applications.
